# Prolonged acute care and post-acute care admission and recovery of physical function in survivors of acute respiratory failure: a secondary analysis of a randomized controlled trial

**DOI:** 10.1186/s13054-017-1791-1

**Published:** 2017-07-21

**Authors:** Anna Neumeier, Amy Nordon-Craft, Dan Malone, Margaret Schenkman, Brendan Clark, Marc Moss

**Affiliations:** 1 Division of Pulmonary Sciences and Critical Care Medicine, Department of Medicine, University of Colorado School of Medicine, Research 2, Box C272, 12700 East 19th Avenue, Aurora, CO 80045 USA; 2 Physical Therapy Program, University of Colorado School of Medicine, Aurora, CO USA

**Keywords:** Chronic critical illness, Persistent critical illness, Prolonged hospitalization, Functional recovery, Physical rehabilitation, Acute respiratory failure, Post-acute care

## Abstract

**Background:**

The proportion of survivors of acute respiratory failure is growing; yet, many do not regain full function and require prolonged admission in an acute or post-acute care facility. Little is known about their trajectory of functional recovery. We sought to determine whether prolonged admission influenced the trajectory of physical function recovery and whether patient age modified the recuperation rate.

**Methods:**

We performed a secondary analysis of a randomized clinical trial of intensive physical therapy for patients with acute respiratory failure requiring mechanical ventilation for ≥4 days. The primary outcome was Continuous Scale Physical Functional Performance, short form (CS-PFP-10), score. Predictor variables included prolonged admission in an acute or post-acute care facility at 1 month, time, and patient age. To determine whether the association between admission and functional outcome varied over time, a multivariable mixed effects linear regression model was fit using an interaction between prolonged admission and time with a primary outcome of total CS-PFP-10 score.

**Results:**

Of the 89 patients included, 56% (50 of 89) required prolonged admission. At 1 month, patients who remained admitted had CS-PFP-10 scores that were 20.1 (CI 10.4–29.8) points lower (*p* < 0.0001) than patients who were discharged to home. However, there was no difference in the rate at which physical function improved from 3 to 6 months for patients who required prolonged admission compared with those who returned home (*p* = 0.24 for interaction between prolonged admission and time). Adjusted for age, Acute Physiology and Chronic Health Evaluation II score, and sex, both groups had CS-PFP-10 scores that were 8.2 (CI 4.5–12.0) points higher at 6 months than at 3 months (*p* < 0.0001). For each additional year in patient age, CS-PFP-10 recovered 0.36 points slower (95% CI 0.12–0.61; *p* = 0.004).

**Conclusions:**

Patients who require prolonged admission after acute respiratory failure have significantly lower physical functional performance than patients who return home. However, the rates of physical functional recovery between the two groups do not differ. The majority of survivors do not recover sufficiently to achieve functional independence by 6 months. Older age negatively influences the trajectory of functional recovery.

**Trial registration:**

ClinicalTrials.gov, NCT01058421. Registered on 26 January 2010.

**Electronic supplementary material:**

The online version of this article (doi:10.1186/s13054-017-1791-1) contains supplementary material, which is available to authorized users.

## Background

With advances in intensive care, survival of patients with acute respiratory failure has improved. Functional impairments in these survivors are common and persist up to 5 years after the initial episode of illness [1]. As a result, there is a growing proportion of patients who do not fully recover and remain dependent on hospital resources. These chronically critically ill patients have high mortality, and those who survive have functional and cognitive disabilities [2–4]. The rate of post-acute care admission in survivors of acute respiratory failure continues to rise, with a greater proportion of patients requiring extended treatment in a long-term acute care facility, rehabilitation facility, or skilled nursing facility and fewer patients returning directly home [5, 6]. Unfortunately, interventions to treat and improve physical function have had variable efficacy, and to date, randomized controlled studies of both intensive inpatient and outpatient physical therapy interventions as well as multidisciplinary approaches designed to increase physical function have not improved long-term physical function [7–12].

Although prolonged admission is common in patients with chronic critical illness, little is known about the long-term trajectory of functional recovery for those patients who survive. Understanding how their recovery unfolds over time and the factors that influence this recovery could inform the timing and intensity of future therapeutic physical interventions; help in the design of unique therapies for specific patient populations; and provide prognostic information to inform patients and their families about the likelihood, rate, and anticipated course of functional recovery. Prior efforts to identify predictors of functional recovery have been focused on patient characteristics prior to acute illness or on intensive care unit (ICU)-related factors [13, 14]. Although equally important, to date, there has been limited evaluation of patient predictors of long-term functional recovery based on functional status after acute illness. Furthermore, because older patients are more likely to have loss of physical function after hospital discharge [13–15], understanding how age influences the trajectory of recovery could highlight a group at particularly high risk of poor outcomes.

To further understand functional recovery over time in patients who require prolonged admission, we performed a secondary analysis of a previously published exercise intervention study [12]. We defined prolonged admission as requiring ongoing admission at 1 month after critical illness in an acute or post-acute care facility, such as a long-term acute care hospital, skilled nursing facility, or rehabilitation facility. Because the availability of post-acute care facilities such as long-term acute care facilities and skilled nursing facilities is variable across national and international healthcare systems, many of the patients in this U.S. population cohort would otherwise remain hospitalized and therefore were included in the prolonged admission group. We sought to determine whether the magnitude of the association between time and physical function varied on the basis of whether a patient required prolonged admission or returned home. Furthermore, we sought to determine whether older patients recover physical function at a lower rate. Understanding the association between functional recovery and age could identify groups who require more intensive and long-term therapeutic interventions to improve functional outcomes.

## Methods

### Study setting and sample

We performed a secondary analysis of a multicenter randomized clinical trial evaluating the efficacy of an intensive physical therapy program compared with usual care [12]. In the parent study, adults with respiratory failure requiring mechanical ventilation for ≥4 days were included. Patients with acute myocardial infarction, aortic dissection, or pulmonary embolism were excluded primarily for safety concerns. Additionally, patients who were unable to participate in physical therapy because of language barriers or cognitive impairments, as well as patients whose anticipated survival was <6 months, were excluded from the trial. The parent study demonstrated no significant difference in outcomes between intensive physical therapy and usual care.

### Outcome variables

Physical function measured by the score on the Continuous Scale Physical Functional Performance, short form (CS-PFP-10), was assessed at 1, 3, and 6 months among survivors who were not in an acute or post-acute care facility. The CS-PFP-10 is an objective, performance-based measure of activities of daily living [16]. The individual is assessed on the basis of ability to perform ten activities of daily living. These tasks range from low difficulty (e.g., putting on a jacket, pouring water from a jug into a cup) to high difficulty (e.g., carry groceries 70 m, climbing stairs). The tasks contribute to a CS-PFP-10 total score as well as to five separate domain subscales, including upper extremity strength, upper extremity flexibility, lower extremity strength, balance and coordination, and endurance. All scales, including the total score, are scored from 0 to 100. A total CS-PFP-10 score of 57 is an accepted threshold below which the probability of living independently is significantly reduced [17]. The primary outcome variable was the CS-PFP-10 score over time, treated as a continuous variable.

### Predictors of interest

Prolonged admission was assessed as a categorical variable and was defined as ongoing admission in an acute care hospital, long-term acute care hospital, or skilled nursing facility 1 month after acute critical illness and the time of randomization in the clinical trial. Because patients who were admitted at 1 month were not assessed for physical functional performance testing, only the time period between 3 and 6 months was assessed for analysis. Time was assessed as a categorical variable (3 months after study randomization vs 6 months after study randomization). To evaluate whether the magnitude of the association between prolonged admission and physical recovery varied over time, we considered an interaction term between prolonged admission and time. To evaluate whether the association between time and recovery of physical function depended on age, we also considered an interaction term between time and age in a separate multivariable model (considered as a continuous variable).

### Covariates

Variables potentially associated with physical function were considered as potential confounding variables. These included severity of illness as measured by the Acute Physiology and Chronic Health Evaluation II (APACHE II) score, as well as male sex.

### Statistical analysis

Differences between patients requiring prolonged admission and those who returned home were assessed using the Wilcoxon rank-sum test or Student’s *t* test, as appropriate, for continuous variables and Pearson’s chi-square test for categorical variables. To determine whether the association between time and physical function varied on the basis of prolonged admission, we used a multivariable mixed effects linear regression model with an interaction term between prolonged admission and time as the predictor of interest and total CS-PFP-10 score as the outcome of interest. This model was adjusted for age, sex, and APACHE II score. To account for correlated data, random slopes and intercepts were included for subjects. Secondary analyses included identical multivariable regression models using the subscales of CS-PFP-10 as the outcome variable. A separate sensitivity analysis was performed that excluded patients who did not undergo CS-PFP-10 testing at 3 and 6 months. To test whether the association between time and physical function was dependent on age, we used a similar mixed effects multivariable linear regression model with an interaction between time and age as the predictor of interest and total CS-PFP-10 as the outcome variable. This model was adjusted for APACHE II, sex, and prolonged admission. The primary inference for all multivariable models was based on the *p* value of the first-order interaction term, with *p* < 0.05 considered statistically significant.

## Results

**Fig. 1:**
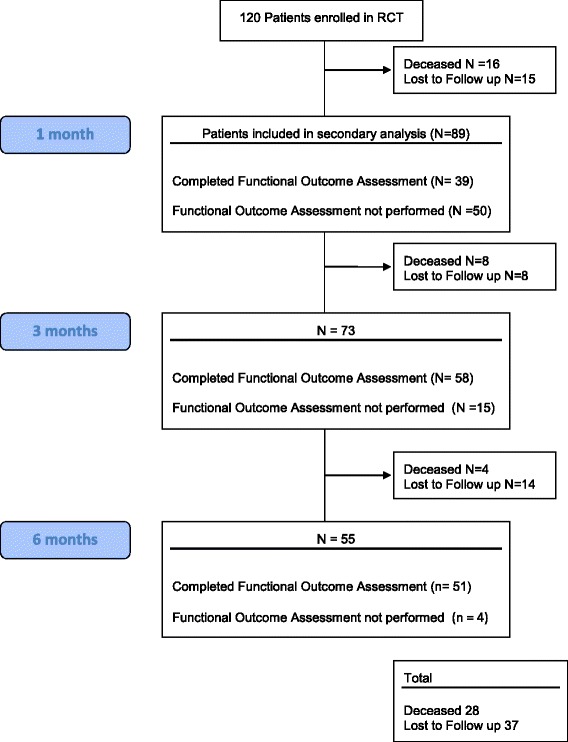
Consolidated Standards of Reporting Trials (CONSORT) diagram of secondary analysis. *RCT* Randomized controlled trial

Of the 120 patients enrolled in the clinical trial, 28 died during the 6-month follow-up period. A total of 89 patients were included in the secondary analysis (Fig. 1). At 1 month, 86% (89 of 104) of survivors had follow-up. At 3 and 6 months, 76% (73 of 96) and 60% (55 of 92) of survivors, respectively, had follow-up. At 1 month, functional outcome testing and CS-PFP-10 testing were not assessed in 56% (50 of 89) of survivors, owing to prolonged admission in an acute or post-acute care facility. At 3 and 6 months, functional outcomes were not assessed in 20% (15 of 73), and 7% (4 of 55), respectively, owing to ongoing admission in an acute or post-acute care facility.

The mean age was 52 ± 15 years, 59% were male, and the mean APACHE II score was 17.6 ± 5.7. Common diagnoses were acute respiratory distress syndrome (32%), nonpulmonary sepsis (22%), and pneumonia (21%). The mean total CS-PFP-10 score at 1 month was 20 ± 26 (*n* = 89). At 3- and 6-month follow-up, the mean total CS-PFP-10 scores were 35 ± 26 (*n* = 73) and 42 ± 24 (*n* = 55), respectively. Patients who required prolonged admission at 1 month compared with those who returned home were older (mean age 55 vs. 49 years; *p* = 0.04) and were more likely to require ongoing admission in a post-acute care facility at 3 and 6 months (Table 1). In comparison of baseline demographics, we observed that compared with those patients who were included in the secondary analysis, patients who were lost to follow-up at 1 month had lower APACHE II scores (14.6 ± 4.6 vs. 17.5 ± 5.8; *p* = 0.04) and those who were lost to follow-up at 6 months were younger (48 ± 14 vs. 54 ± 14 years; *p* = 0.05) (Table 2).

**Table 1:** Baseline characteristics

	Entire cohort (*n* = 120)	Prolonged admission (*n* = 50)	Discharged to home at 1 month (*n* = 39)	*p* Value
Age, years	55 ± 15	55 ± 16	49 ± 14	0.04
Male sex, *n* (%)	71 (59%)	29 (58%)	23 (59%)	1
Race or ethnicity, *n* (%)				
Caucasian	93 (77%)	37 (74%)	32 (82%)	0.4
Other	27 (23%)	13 (26%)	7 (18%)	0.4
APACHE II score	18 ± 6	18 ± 5	17 ± 6	0.2
Diagnoses				
ALI/ARDS	35 (29%)	14 (28%)	15 (38%)	0.6
PNA/aspiration	39 (33%)	15 (30%)	10 (25%)	0.6
Other	46 (38%)	21 (42%)	14 (35%)	0.6
Intensive physical therapy treatment received	59 (49%)	21 (42%)	20 (51%)	0.7
Comorbidities				
Cancer, *n* (%)	10 (9%)	4 (8%)	2 (5%)	0.6
Diabetes, *n* (%)	27 (23%)	13 (26%)	6 (15%)	0.3
Cirrhosis, *n* (%)	16 (13%)	8 (16%)	3 (8%)	0.3
Renal failure, *n* (%)	8 (7%)	1 (3%)	3 (6%)	0.7
HIV, *n* (%)	4 (3%)	1 (2%)	2 (5%)	0.6
Received corticosteroids, *n* (%)	21 (17%)	10 (20%)	6 (15%)	0.8
Acute care hospital LOS, days	21 (14–33)	30 (21–45)	16 (12–26)	<0.0001
Initial discharge location				
Home	52 (43%)	9 (18%)	31 (79%)	<0.0001
Post-acute care facility	50 (42%)	38 (76%)	8 (21%)	<0.0001
Prolonged admission at 3 months, *n* (%)	15 (21%)	14 (38%)	1 (6%)	0.0016
Prolonged admission at 6 months, *n* (%)	4 (7%)	4 (15%)	0	0.034

*Abbreviations: ALI* Acute lung injury, *APACHE II* Acute Physiology and Chronic Health Evaluation II, *ARDS* Acute respiratory distress syndrome, *LOS* Length of stay, *PNA* Pneumonia

**Table 2:** Missing data analysis

	1 month			3 months			6 months		
Patient characteristics	Follow-up (*n* = 89)	Lost to follow-up (*n* = 15)	*p* Value	Follow-up (*n* = 73)	Lost to follow-up (*n* = 23)	*p* Value	Follow-up (*n* = 55)	Lost to follow-up (*n* = 37)	*p* Value
Age, years	52 ± 15	51 ± 15	0.7	53 ± 15	49 ± 14	0.2	54 ± 14	48 ± 14	0.05
Male sex, *n* (%)	51 (57%)	10 (63%)	0.7	42 (58%)	16 (69%)	0.3	32 (58%)	24 (65%)	0.5
Caucasian race, *n* (%)	68 (77%)	11 (68%)	0.4	53 (73%)	19 (83%)	0.3	41 (75%)	28 (76%)	0.9
APACHE II score	17.5 ± 5.8	14.6 ± 4.6	0.04	17.8 ± 5.8	15.6 ± 5.3	0.1	17.4 ± 5.7	17.2 ± 6.2	0.8
Received intensive physical therapy, *n* (%)	40 (45%)	9 (56%)	0.4	34 (47%)	12 (52%)	0.6	28 (51%)	16 (43%)	0.5

*APACHE II* Acute Physiology and Chronic Health Evaluation II

*Total deceased at 1 month (*n* = 15), at 3 months (*n* = 24), and at 6 months (*n* = 28). Deceased patients were excluded from missing data analysis

**Fig. 2:**
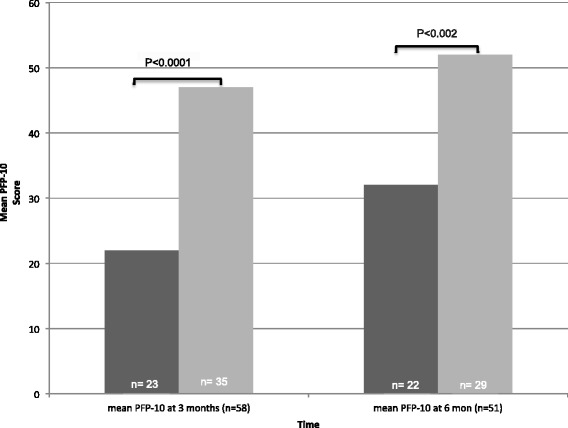
Mean Continuous Scale Physical Functional Performance, short form (PFP-10), scores over time in patients with prolonged admission compared with patients who returned home at 1 month. *Dark gray* = prolonged admission (*n* = 50); *light gray* = discharged to home at 1 month (*n* = 39)

In both groups, significant physical function limitations were present 6 months after recovery (mean CS-PFP-10 = 43 [CI 36–49]). Compared with patients who had returned home, patients who required prolonged admission had significantly lower physical function at 3 months (mean total CS-PFP-10 = 22 vs. 47; *p* < 0.0001) and 6 months (mean total CS-PFP-10 = 32 vs. 52; *p* < 0.002) (Fig. 2). When we examined the subscales of the CS-PFP-10 over time, we observed significant differences in functional scores for all functional domains (Table 2). A higher proportion of patients who required prolonged admission at 1 month had died at 6 months than among those who had returned home (22% vs. 2.5%; *p* = 0.007).

Adjusting for age, sex, and APACHE II score, we observed no difference in the rate at which physical function improved from 3 to 6 months for patients who required prolonged admission at 1 month compared with those who were discharged to home (*p* = 0.24 for interaction between time and prolonged admission). On average, patients with prolonged admission had CS-PFP-10 scores that were 20.1 (CI 10.4–29.8) points lower (*p* < 0.0001) than those of patients who had been discharged to home at 1 month (Table 3). Both groups had CS-PFP-10 scores that were 8.2 (CI 4.5–12.0) points higher at 6 months than at 3 months (*p* < 0.0001). Male patients had CS-PFP-10 scores that were 7.25 (CI 2.4–12.1) points higher than females. The results were similar with identical multivariable regression models using the five subscales of CS-PFP-10 as the outcome variable. The results of the sensitivity analysis excluding patients with CS-PFP-10 scores of 0 at 3 and 6 months were similar without a significant change in parametric estimates (Additional files 1 and 2).

**Table 3:** Mean Continuous Scale Physical Functional Performance, short form, total and subscale scores over time

	CS-PFP-10 in patients with prolonged admission	CS-PFP-10 in patients discharged to home at 1 month	*p* Value
CS-PFP-10 total score (3 months)	22 (15–29)	47 (38–55)	<0.0001
CS-PFP-10 total score (6 months)	32 (23–42)	52 (44–60)	<0.002
CS-PFP-10 subscales			
Upper body strength (3 months)	21 (13–29)	50 (41–59)	<0.0001
Upper body strength (6 months)	36 (25–46)	53 (42–63)	0.02
Lower body strength (3 months)	16 (9–23)	40 (33–47)	<0.0001
Lower body strength (6 months)	25 (16–34)	43 (34–51)	0.005
Balance/coordination (3 months)	22 (15–30)	47 (39–55)	<0.0001
Balance/coordination (6 months)	32 (23–41)	52 (44–61)	0.002
Endurance (3 months)	22 (14–30)	47 (39–55)	<0.0001
Endurance (6 months)	32 (23–41)	52 (44–61)	0.001
Flexibility (3 months)	38 (29–48)	63 (52–73)	0.0012
Flexibility (6 months)	52 (43–61)	73 (64–82)	0.0027

*CS-PFP-10* Continuous Scale Physical Functional Performance, short form

Data in table are presented as means with 95% CIs in parentheses.

The CS-PFP-10 total and component subscales are scored from 0 to 100, with higher scores indicating better function. For adults without disabilities aged 35–54 years, average total CS-PFP-10 scores ranged from 70.9 to 73.9

Patients with functional outcome assessments completed: prolonged admission group: 3 months (*n* = 23), 6 months (*n* = 22); discharged to home at 1 month: 3 months (*n* = 35), 6 months (*n* = 29)

The age of the patient was associated with a slower rate of improvement in physical function with a change in CS-PFP-10 of −0.36 for every increasing year age (CI −0.61 to −0.12; *p* = 0.004 for age × time interaction) (Table 4).

**Table 4:** Association between time and physical function with prolonged admission and patient age (adjusted analysis)

	CS-PFP-10	CI (lower 95%)	CI (upper 95%)	*p* Value
Prolonged admission				
Prolonged admission	**−20.31**	**−29.99**	**−10.63**	**<0.0001**
Time (3–6 months)	**8.20**	**4.40**	**11.90**	**<0.0001**
Age	**−0.37**	**−0.71**	**−0.05**	**0.026**
APACHE II	−0.71	−1.52	0.11	0.08
Male sex	**7.25**	**2.43**	**12.07**	**<0.0038**
Time × prolonged admission interaction	4.48	−3.02	11.98	0.24
Age				
Age	**−0.40**	**0.73**	**−0.07**	**0.019**
Time (3–6 months)	**8.30**	**4.86**	**11.84**	**<0.001**
Prolonged admission	**−20.24**	**−30.01**	**−10.45**	**0.0001**
APACHE II	−0.70	−1.52	0.11	0.09
Male sex	**7.25**	**2.38**	**12.11**	**0.004**
Age × time interaction	**−0.36**	**−0.61**	**−0.12**	**0.004**

*Abbreviations: APACHE II* Acute Physiology and Chronic Health Evaluation II, *CS-PFP-10* Continuous Scale Physical Functional Performance, short form

*Prolonged admission, categorical variable, defined as persistent admission to acute care hospital or post-acute care facility (long-term acute care facility, skilled nursing facility, or rehabilitation facility) at 1 month after study enrollment; time, categorical variable (period from 3 to 6 months); age, continuous variable; APACHE II, continuous variable; male sex, categorical variable

## Discussion

This secondary analysis demonstrates that in patients recovering from respiratory failure requiring mechanical ventilation, physical functional limitations are common. Prior to illness, all patients were living independently; yet, by 6 months, few had regained such function. The mean CS-PFP-10 score achieved by patients at 6 months was only 42, well below the threshold score of 57 that is associated with the ability to live independently [16, 17]. Compared with patients who returned home, patients who required prolonged admission had significantly lower physical function even by 6 months of recovery after an episode of acute respiratory failure (CS-PFP-10 = 32 vs. 55). Despite likely receiving higher-intensity rehabilitative services, patients who required prolonged admission were unable to achieve physical function levels of those patients who had returned home. Interestingly, both groups demonstrated parallel trajectories of recovery to those of patients who required prolonged admission and demonstrated improvement in physical function between 3 and 6 months of recovery at a rate similar to those who had returned home.

Our results suggest that patient heterogeneity may influence functional recovery after critical illness and that risk stratification based on ongoing need for admission in an acute or post-acute care setting at 1 month after an episode of acute respiratory failure may feasibly predict long-term physical functional recovery and guide rehabilitation interventions. Because patients respond differently to physical therapy, a single prescriptive approach for all patients may be ineffective and may contribute to findings from the numerous clinical trials of intensive physical therapy that have not demonstrated benefit in improving long-term functional recovery [7, 9, 11]. Instead, a targeted rehabilitation approach directed at patient subgroups that is based on patient-specific predictors has been hypothesized to be more effective. In a recent secondary analysis, Puthucheary and Denehy demonstrated that patient characteristics, specifically the presence or absence of preexisting chronic disease, were associated with trajectory of functional recovery [14]. Our secondary analysis suggests that age as well as prolonged admission after an episode of acute respiratory failure may also serve as predictors of future long-term recovery. Because younger age has been shown on the basis of observational data to be associated with a greater rate of functional recovery [1], it seems likely that age influences response to therapy. Recently, Ferrante and colleagues demonstrated that, in older adults aged 70 years and older who survived an ICU hospitalization, only 52.3% had recovery of pre-ICU function [15]. Therefore, older patients may need a different intensity or form of therapy to achieve rates of recovery similar to those of younger age groups. Additionally, in our model, there was an association between male sex and greater functional recovery. Gender differences in mobility have previously been observed; women have been shown to have greater levels of disability in their mobility [18, 19]. The reasons for this are not well understood but are an important area of further study. Furthermore, if gender is a patient characteristic potentially influential on physical functional recovery, this characteristic would be important in guiding targeted future interventions to promote post-ICU recovery.

Prolonged admission in an acute or post-acute care setting after an episode of respiratory failure may serve as a predictor of recovery or, alternatively, may be associated with the intensity of rehabilitation services received. In the original study, patients who were persistently admitted at 1 month resided either in an acute care facility or in a post-acute care facility (long-term care, skilled nursing, or rehabilitation facility). It is likely that during that 1 month, these patients received a higher intensity of rehabilitative therapy than those who returned home; however, their rate of recovery between 3 and 6 months postillness was similar to that of patients who returned home. This finding raises the possibility that physical functional recovery may be influenced not only by intensity of therapy but also perhaps by timing of physical therapy interventions. Because the majority of patients in both groups continued to have functional limitations even at 6 months postillness, it appears that physical functional recovery plateaus. One possible explanation for this includes that, upon returning home, survivors of acute respiratory failure receive limited physical functional rehabilitation services. Patients likely need a longer duration of therapy beyond what is provided in the hospital, and ongoing rehabilitation at home may similarly need to be intensified.

Our study has several strengths. By use of a performance-based outcome measure of physical function, the functional limitations of these survivors can be objectively measured. With the CS-PFP-10 assessment, we were able to define functional limitations in terms of a survivor’s ability to perform activities of daily living, quantify the severity of their impairments, and objectively describe their functional recovery over time. Furthermore, in this relatively young cohort, our demonstration that age influences functional recovery is an important finding. Last, we demonstrate that although prolonged admission is associated with lower physical functional performance, these patients do recover physical function over time. Stratification based on prolonged admission could be used as a potential predictor of long-term functional recovery.

The results of this secondary analysis are limited by those lost to follow-up as well as those unable to complete functional outcome assessments. Patients who were lost to follow-up at 1 month had higher baseline APACHE II scores than those included in the cohort, but by 6 months the baseline APACHE II scores were not different. By 6 months, those lost to follow-up were slightly younger than the entire cohort. Therefore, it is possible that if younger, healthier patients were lost to follow-up, the functional recovery in this cohort would be underestimated. Our analysis is also limited by those patients who did have functional outcome assessments because of ongoing admission in an acute or post-acute care facility. Because many patients required prolonged admission at 1 month and were therefore assigned a CS-PFP-10 score of 0, the 1-month functional outcome analysis was excluded. Thus, the initial early change in function between 1 and 3 months cannot be fully characterized. Additionally, this also may underpower the overall analysis. Additionally, we could not adjust for unmeasured confounding variables such as intensity of physical therapy received outside the clinical trial, which may have influenced changes in functional performance testing.

## Conclusions

Our results suggest that physical functional limitations in survivors of acute respiratory failure requiring mechanical ventilation are common. This analysis is unique in that the level of physical functional recovery was quantified over time using the CS-PFP-10, a robust, performance-based measure of physical functional ability in multiple domains, as opposed to single-domain physical function assessments or questionnaire-based data. Furthermore, the results offer additional insight into physical functional recovery of patients who require prolonged admission in an acute or post-acute care setting. Although both groups recover, those who remain hospitalized 1 month after critical illness do not achieve similar levels of physical function by 6 months, and neither group achieves physical function associated with functional independence. Age negatively influences this trajectory of recovery. Further research is needed to intensify and individualize therapy to promote functional recovery as patients transition from the hospital and from the post-acute care setting to home.

## Additional files

Additional file 1: Sensitivity analysis 7.5.17. This is a sensitivity analysis of the multivariable linear regression model excluding patients with PFP-10 scores of 0 at 3 and 6 months. (DOCX 70 kb)

Additional file 2: Initial discharge location. This describes the initial discharge (home, skilled nursing facility, long-term acute care, rehabilitation facility, hospice, deceased) of the two groups of patients. (DOCX 49 kb)

## Abbreviations

ALI: Acute lung injury
APACHE II: Acute Physiology and Chronic Health Evaluation II
ARDS: Acute respiratory distress syndrome
CONSORT: Consolidated Standards of Reporting Trials
CS-PFP-10: Continuous Scale Physical Functional Performance, short form
ICU: Intensive care unit
LOS: Length of stay
RCT: Randomized controlled trial

## References

[1] Herridge MS, Tansey CM, Matté A, Tomlinson G, Diaz-Granados N, Cooper A (2011). Functional disability 5 years after acute respiratory distress syndrome. N Engl J Med.

[2] Kahn JM, Le T, Angus DC, Cox CE, Hough CL, White DB (2015). The epidemiology of chronic critical illness in the United States. Crit Care Med.

[3] Nelson JE, Cox CE, Hope AA, Carson SS (2010). Chronic critical illness. Am J Respir Crit Care Med.

[4] Nelson JE, Tandon N, Mercado AF, Camhi SL, Ely EW, Morrison RS (2006). Brain dysfunction: another burden for the chronically critically ill. Arch Intern Med.

[5] Kahn JM, Benson NM, Appleby D, Carson SS, Iwashyna TJ (2010). Long-term acute care hospital utilization after critical illness. JAMA.

[6] Makam AN, Nguyen OK, Zhou J, Ottenbacher KJ, Halm EA (2015). Trends in long-term acute care hospital use in Texas from 2002–2011. Ann Gerontol Geriatr Res.

[7] Walsh TS, Salisbury LG, Merriweather JL, Boyd JA, Griffith DM, Huby G (2015). Increased hospital-based physical rehabilitation and information provision after intensive care unit discharge: the RECOVER randomized clinical trial. JAMA Intern Med.

[8] Cuthbertson BH, Rattray J, Campbell MK, Gager M, Roughton S, Smith A (2009). The PRaCTICaL study of nurse led, intensive care follow-up programmes for improving long term outcomes from critical illness: a pragmatic randomised controlled trial. BMJ.

[9] Denehy L, Skinner EH, Edbrooke L, Haines K, Warrillow S, Hawthorne G (2013). Exercise rehabilitation for patients with critical illness: a randomized controlled trial with 12 months of follow-up. Crit Care.

[10] Connolly B, Salisbury L, O’Neill B, Geneen L, Douiri A, Grocott MP (2015). Exercise rehabilitation following intensive care unit discharge for recovery from critical illness. Cochrane Database Syst Rev.

[11] Elliott D, McKinley S, Alison J, Aitken LM, King M, Leslie GD (2011). Health-related quality of life and physical recovery after a critical illness: a multi-centre randomised controlled trial of a home-based physical rehabilitation program. Crit Care.

[12] Moss M, Nordon-Craft A, Malone D, Van Pelt D, Frankel SK, Warner ML (2016). A randomized trial of an intensive physical therapy program for patients with acute respiratory failure. Am J Respir Crit Care Med.

[13] Needham DM, Wozniak AW, Hough CL, Morris PE, Dinglas VD, Jackson JC (2014). Risk factors for physical impairment after acute lung injury in a national, multicenter study. Am J Respir Crit Care Med.

[14] Puthucheary ZA, Denehy L (2015). Exercise interventions in critical illness survivors: understanding inclusion and stratification criteria. Am J Respir Crit Care Med.

[15] Ferrante LE, Pisani MA, Murphy TE, Gahbauer EA, Leo-Summers LS, Gill TM (2016). Factors associated with functional recovery among older intensive care unit survivors. Am J Respir Crit Care Med.

[16] Cress ME, Petrella JK, Moore TL, Schenkman ML (2005). Continuous-Scale Physical Functional Performance Test: validity, reliability, and sensitivity of data for the short version. Phys Ther.

[17] Cress ME, Meyer M (2003). Maximal voluntary and functional performance levels needed for independence in adults aged 65 to 97 years. Phys Ther.

[18] Leveille SG, Penninx BW, Melzer D, Izmirlian G, Guralnik JM (2000). Sex differences in the prevalence of mobility disability in old age: the dynamics of incidence, recovery, and mortality. J Gerontol B Psychol Sci Soc Sci.

[19] Mechakra-Tahiri SD, Freeman EE, Haddad S, Samson E, Zunzunegui MV (2012). The gender gap in mobility: a global cross-sectional study. BMC Public Health.

