# Supplementary material for: Prolonged acute care and post-acute care admission and recovery of physical function in survivors of acute respiratory failure: a secondary analysis of a randomized controlled trial
Source: Crit Care. 2017 Jul 21;21:190. doi: 10.1186/s13054-017-1791-1 (PMC5521116; doi:10.1186/s13054-017-1791-1)
Supplement: Supplementary file 1 — Sensitivity analysis 7.5.17. This is a sensitivity analysis of the multivariable linear regression model excluding patients with PFP-10 scores of 0 at 3 and 6 months. (DOCX 70 kb) [file 13054_2017_1791_MOESM1_ESM.docx]

| **A) Prolonged Admission** | | | | |
| --- | --- | --- | --- | --- |
|  | PFP-10 | CI (lower 95%) | CI (upper 95%) | P |
| Prolonged Admission | **-39.32** | **-50.17** | **-28.6** | **<0.0001** |
| Time (3-6 months) | **4.56** | **2.23** | **6.9** | **0.0003** |
| Age | **-0.33** | **-0.65** | **-0.004** | **0.04** |
| APACHE II | **-0.87** | **-1.67** | **-0.06** | **0.03** |
| Gender (male) | **5.7** | **0.66** | **10.3** | **0.03** |
| Time*Prolonged Admission Interaction | 3.65 | -1.1 | 8.42 | 0.12 |

Additional Data: Sensitivity Analysis:

*Multivariable mixed effects linear regression model excluding patients with PFP-10 scores of 0 at 3 months (n=14) and 6 months (n=4).

*Prolonged admission- categorical variable, defined as persistent admission to acute-care hospital or post-acute care facility (long-term acute care facility, skilled-nursing facility, or rehabilitation facility at one month after study enrollment; Time: categorical variable (3-6 month period); age: continuous variable, APACHE II: continuous variable, Gender: male, categorical variable
