# Supplementary material for: Prolonged acute care and post-acute care admission and recovery of physical function in survivors of acute respiratory failure: a secondary analysis of a randomized controlled trial
Source: Crit Care. 2017 Jul 21;21:190. doi: 10.1186/s13054-017-1791-1 (PMC5521116; doi:10.1186/s13054-017-1791-1)
Supplement: Supplementary file 2 — Initial discharge location. This describes the initial discharge (home, skilled nursing facility, long-term acute care, rehabilitation facility, hospice, deceased) of the two groups of patients. (DOCX 49 kb) [file 13054_2017_1791_MOESM2_ESM.docx]

Additional File 2: Initial Discharge Location

| Initial Discharge Location | Cohort | Prolonged Hospitalization at 1 month | Discharged Home at 1 month | P value |
| --- | --- | --- | --- | --- |
|  |  |  |  |  |
| Home | 52 (43%) | 9 (18%) | 31 (79%) | p<0.0001 |
| LTAC | 23 (19%) | 20 (40%) | 3 (8%) | p<0.0001 |
| SNF | 9 (8%) | 5 (10%) | 4 (10%) | p=0.7 |
| Rehab | 18 (15%) | 13 (26%) | 1 (3%) | p=0.06 |
| Deceased | 16 (13%) | 1 (2%) | 0 | p=0.37 |
| Hospice | 2 (2%) | 2 (4%) | 0 | p=0.2 |
